# Gradual Reduction in Sodium Content in Cooked Ham, with Corresponding Change in Sensorial Properties Measured by Sensory Evaluation and a Multimodal *Machine Vision System*

**DOI:** 10.1371/journal.pone.0137805

**Published:** 2015-09-30

**Authors:** Kirsti Greiff, John Reidar Mathiassen, Ekrem Misimi, Margrethe Hersleth, Ida G. Aursand

**Affiliations:** 1 SINTEF Fisheries and Aquaculture, Trondheim, Norway; 2 Department of Biotechnology, Norwegian University of Science and Technology, Trondheim, Norway; 3 Nofima AS, Ås, Norway; University of Lleida, SPAIN

## Abstract

The European diet today generally contains too much sodium (Na^+^). A partial substitution of NaCl by KCl has shown to be a promising method for reducing sodium content. The aim of this work was to investigate the sensorial changes of cooked ham with reduced sodium content. Traditional sensorial evaluation and objective multimodal *machine vision* were used. The salt content in the hams was decreased from 3.4% to 1.4%, and 25% of the Na^+^ was replaced by K^+^. The salt reduction had highest influence on the sensory attributes salty taste, after taste, tenderness, hardness and color hue. The multimodal machine vision system showed changes in lightness, as a function of reduced salt content. Compared to the reference ham (3.4% salt), a replacement of Na^+^-ions by K^+^-ions of 25% gave no significant changes in WHC, moisture, pH, expressed moisture, the sensory profile attributes or the surface lightness and shininess. A further reduction of salt down to 1.7–1.4% salt, led to a decrease in WHC and an increase in expressible moisture.

## Introduction

A high consumption of sodium has been directly associated with a greater likelihood of increased blood pressure, which in turn has been directly related to the development of cardiovascular and renal diseases [1]. For these reasons, national and international bodies have set targets for a reduction in sodium consumption down to 2 g/day [2–4].

Due to the low fat content, cooked ham is a good source of animal protein in the diet. Usually when manufacturing cooked ham, the pork meat is coarsely ground into pieces that are tumbled with brine (containing water, salt, nitrite and other ingredients such as phosphate and polysaccharides). During the tumbling with brine, the salt- soluble myofibrillar proteins are extracted and they form a network. The network gels during the heating and chilling process [5], thereby solidifying the ham and minimizing cooking loss. Reducing salt contents in cooked ham increases cooking loss [6] and thereby the production costs.

Sodium chloride is added to a wide variety of foods to increase shelf life and palatability (e.g. in soups, meat products, bread, sauces and snacks). When it comes to sensory quality, sodium chloride contributes to saltiness and the overall flavor, while also suppressing bitterness [7]. Accordingly, the development of palatable sodium-reduced products is important in order to guide consumers towards more healthy food choices. Partial substitution of NaCl by KCl has shown to be one of the best alternatives for reducing sodium content [5, 8]. Studies have shown that KCl induces a bitter taste at high concentrations, in dry-cured loins [9], fermented sausage[10] and cooked ham [11]. Those results show that the maximum level of KCl replacement may vary among different type of product.

It is essential to characterize and understand the sensory effects of sodium reduction in foods. Sensory analysis is the most appropriate approach to fully describe the sensory perception of foods and descriptive profiling has been a popular sensory technique for cognitive descriptions of products in many years. These techniques give a complete sensory description of products and make it possible to identify underlying ingredient and process variables [12]. For measurement of flavor, no instruments currently exist, that can replace the human senses and describe the sensory perception. For appearance and texture attributes, the situation is different, as there are several examples of studies where instrumental data representing appearance and texture attributes, show a strong correlation with data from trained sensory panels [13–16].

In particular, classification of ham qualities (pork and turkey) is possible based on color and textural features extracted from digital color images. Logistic regression, used on these features, could to a large degree explain consumers' responses for visually-based sensorial attributes [13]. The qualities of pork ham could be distinguished based on visual texture characteristics extracted from fractal analysis of digital color images [16]. This showed that the fractal-based features are able to quantify quality-specific visual texture characteristics. A combination of geometric and texture features, extracted from digital color images, were input to multivariate classifiers, including artificial neural networks (ANNs), and were capable of predicting meat tenderness–as measured by shear force [17]. The eating quality of beef could be predicted from wavelet surface texture and other texture features extracted from digital color images. For the optical setup, a polarizer filter was placed on the lens and the meat pieces were surface-dried to mitigate some specular reflection [14]. Other machine vision approaches have been designed specifically to improve the objective analysis of color and texture in transclucent or semi-transparent muscle foods [18]. These works and others [15, 19] show that computer vision, with optimal illumination in combination with appropriate feature extraction and classifiers, can be used to predict sensorial properties from digital color images.

The effect of reduced salt/sodium content on cooked ham has previously been investigated by different authors [5, 20, 21], but nobody, to the best of our knowledge, has used the combination of sensorial Descriptive Analysis (DA) and multimodal *machine vision system* to investigate the changes in the sensorial properties in cooked ham with effect of reduced salt/sodium content. In this paper, a proof-of-concept novel dual-polarization multimodal machine vision system is used, that is new to meat imaging [22].

The aim of this study was to investigate sensory quality of cooked pork ham containing gradually reduced salt/sodium content and a partial replacement of sodium (Na^+^) by (K^+^). The product quality parameters addressed were physicochemical and sensory properties obtained by sensory descriptive analyses and a multimodal *machine vision system*.

## Materials and Methods

### Samples and Chemicals

#### Cooked ham preparation

Eight different formulas of cooked hams were manufactured by Espeland AS, Ålgård, Norway 26–28 February 2013. Each of the formulas contained 20 kg of fresh coarsely grounded (perforated disc with hole size 30 mm), lean pork meat (*M*. *semimembranosus*, *M*. *adductor*, *M*. *semitendinosus*, *M*. *biceps femoris and M*. *psoas major)*, obtained from a local meat processing company (PrimaGruppen, Norway) four days after slaughtering. The samples are identified with sample IDs having the following codes: Na100, NaK100, NaK80, NaK60, NaK40, NaK20, which vary in the % of the amount of NaCl relative to the reference (Na100) and addition of potassium (NaK). See Table 1 for details. Reference cooked ham (Na100) had no added potassium, containing only potassium naturally occurring in the meat (approximately 330 mg K^+^/100g). The amount of sodium in the reference (Na100) in the formulation was at same level as in a commercially cooked ham, 1.24 g Na^+^/100g, corresponding to 3.1% salt. The mole ratio of sodium (Na^+^) and potassium (K^+^) was kept constant (Na^+^: K^+^ = 3:1) in all formulas containing potassium (NaK), were the contribution of all sodium and potassium in all ingredients were taken into account. The salt (NaCl) content (g/100g) was calculated as: added Na^+^ (including natural sodium content in the meat) x 2.5 (recalculation factor for NaCl from Na^+^), according on the provision of food information to consumers [23]. The amount of total salt content in the formulation was gradually decreased to 20% of the amount of the salt in the reference cooked ham, corresponding to 1.3% salt (NaK20). The brine solution for each formulation was prepared by dissolving 200 g commercial phosphate mixture (57% P_2_O_5_)(A. B. Corneliussen AS, Norway), 150 g gelatin (Gelita Sweden AB, Sweden), 60 g carrageenan (CEAMSA, Pontverda, Spain), 450 g Na-lactate (Purac Biochem, Gorichem, The Netherlands) and 150 g maltodextrin (Avebe U.A., The Netherlands) in 2.5 L tap water (t = 2–4°C). All formulas, excluding the reference ham (Na100), were added 3.3 g sodium nitrite (Merck, KGaA Darmstadt, Germany). Commercial food salt containing nitrite salt (Hoff Norske Potetindustrier, Gjøvik, Norway) was added to the reference ham (Na100). Sodium chloride (Hoff Norske Potetindustrier, Gjøvik, Norway) and potassium chloride (Culinar Lyckeby, Sweden) were added to the brine to achieve the expected Na^+^ and K^+^ content as shown in Table 1.

**Table 1:** Sample ID, description of the sample, estimated sodium, potassium and NaCl content in the preparation of the eight cooked hams. Each formulation varied in its amount of total salt. The reference cooked ham contained nitrite salt (Na 100 Reference). In the rest of the formulas (NaK100, NaK80, NaK60, NaK40 and NaK20) the amount of total salt content was gradually decreased to 20% of the amount of the salt in the reference cooked ham. To keep the mole ratio of sodium (Na^+^) and potassium (K^+^) constant (Na^+^: K^+^ = 3:1), all contribution of sodium and potassium from the raw material and the ingredients was added when calculation of the mole ratio.

Sample ID	Description	Estimated sodium content (g/100 g ham)	Estimated potassium content (g/100 g ham)	NaCl**(%)	% reduction of sodium compared to the reference
Na 100	Reference ham	1.24	0.28	3.1	0
NaK 100_1*	100% of the amount of salt in the reference	1.06	0.61	2.6	14
NaK 100_2*	100% of the amount of salt in the reference	1.06	0.61	2.6	14
NaK 80	80% of the amount of salt in the reference	0.93	0.53	2.3	25
NaK 60_1*	60% of the amount of salt in the reference	0.80	0.46	2.0	35
NaK 60_2*	60% of the amount of salt in the reference	0.80	0.46	2.0	35
NaK 40	40% of the amount of salt in the reference	0.67	0.38	1.7	46
NaK 20	20% of the amount of salt in the reference	0.53	0.31	1.3	57

* Produced twice to control the reproducibility of the production

** The salt content (%) is calculated by using the formula: added sodium (Na^+^), including natural Na^+^ content in the meat x 2.5 (recalculation factor for NaCl from Na^+^)

The brine was added to the pork meat in a tumbler (Fatosa, Barcelona, Spain) without vacuum and massaged for 2 x 25 min, stored 20 h at 2°C, and then tumbled for 15 more min. The meat matrix was stuffed in to a plastic casing (160 mm in diameter, Viscofan S. A., Navarra, Spain). Two parallel production batches of the hams with formulation NaK100 and NaK60 were prepared to control the reproducibility of the production. Seven hams (3.0 kg) from each formulation were produced. The hams were pressed in molds and kept at 0–2°C for 20 h before cooking. The hams were cooked in a cooking chamber (Fessmann, Germany) until they reached a core temperature of 78°C, chilled down in tap water and kept in a cold-storage chamber at 0–2°C. Two of the hams per formula were sliced in 20 slices (16 ± 1 g) 12 days after production, and alternate of the slices were subjected to sensorial evaluation and analysis of machine vision, respectively. Sensorial evaluation and machine vision analyses were carried out directly after slicing. The remaining cooked hams were stored at 4°C for 15 days after production before chemical analysis. In addition, one slice (approximately 100 g) was used for further analysis.

#### Chemicals

Ammonium chloride (NH_4_Cl), ammonium hydroxide (NH_4_OH) and ammonium hydrogen fluoride (NH_4_FHF < 1%, LD_50_ mg/kg not found) of analytical-reagent grade (Thermo Fisher Scientific, USA). Chemicals of food grade: sodium tripoly phosphate (Na_5_P_3_O_10_) (A. B. Corneliussen AS, Norway), Na-lactate (C_3_H_5_NaO_3_) (Purac Biochem, Gorichem, The Netherlands) and sodium nitrite (NNaO_2_)(Merck, KGaA Darmstadt, Germany).

### Imaging Setup and Image Acquisition

The imaging system consisted of a ColorRanger multimodal line-scan camera (SICK IVP AB, Linköping, Sweden), two high-intensity white LED linear array lights (Banner Engineering, Minneapolis, MN, USA), with polarizers on the LED arrays and in front of the camera lens. Additionally, a nematic liquid crystal industrial-grade polarization rotator (ARCoptix SA, Neuchâtel, Switzerland) was placed in front of the polarizer on the camera lens. The polarization rotator is controlled by the image acquisition PC and enables effectively to rotate the polarizer on the lens by means of an electric signal. The purpose of rotating the polarizer direction on the lens polarizer relative to the polarizer on the LEDs, was to be able to capture light reflected from the object for two polarization states.

**Fig 1:**
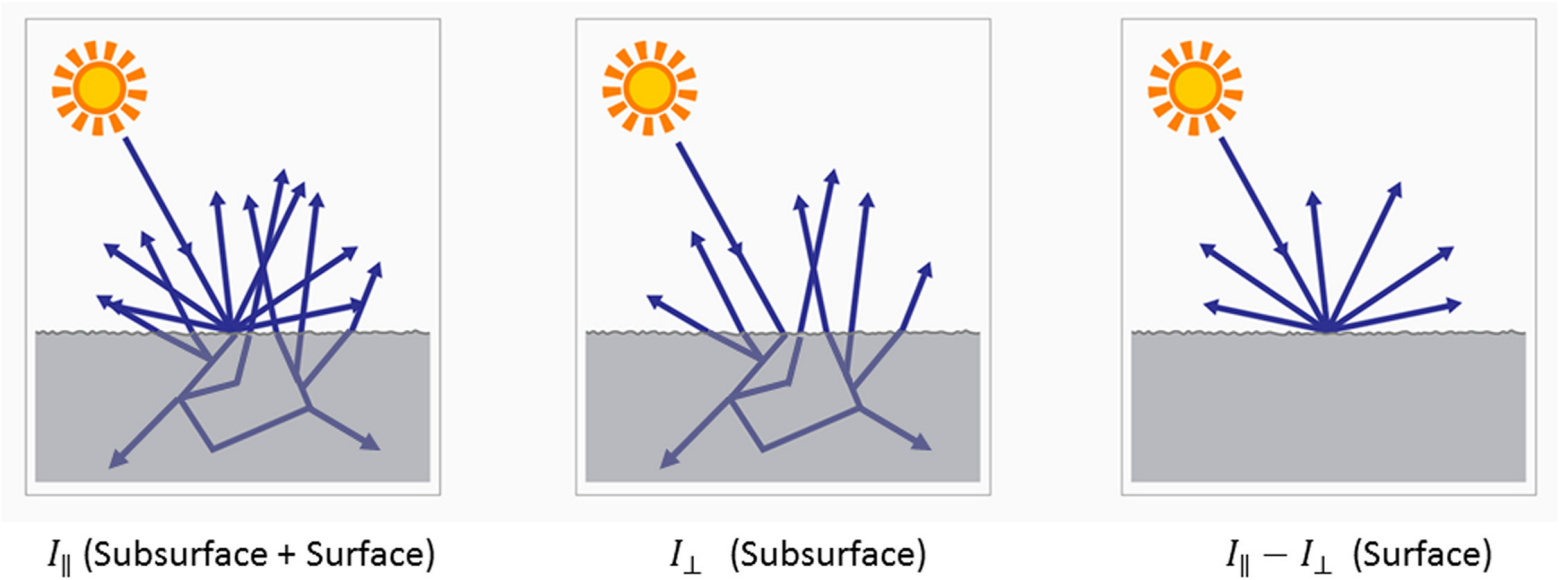
Illustration of the components of light interacting with the imaged raw material, imaged using parallel polarizers (left), crossed polarizers (middle) and the difference between the two (right).

The experiment sought to separately image the subsurface color and the surface color of the ham slices, in order to separately image the light that was scattered in the subsurface from the light that was directly reflected off the surface. Subsurface imaging revealed the bulk color of the ham near the surface, whereas surface imaging revealed the surface roughness, shininess and other effects such as "mother-of-pearl" appearance commonly seen in some hams. To separate the subsurface from the surface image, two images were acquired–an image *I*_∥_ with LED and camera polarizers oriented parallel to each other, and an image *I*_⊥_ with the polarizers oriented perpendicular to each other. Image *I*_∥_ will image both the subsurface and the surface components, whereas *0049*_⊥_ will image the subsurface components only, and hence the difference *I*_∥_—*I*_⊥_ between the two images will image only the surface components of the light. This principle of light interactions as a function of polarization state is illustrated in images in Fig 1.

The extracted imaging features were simply the mean of the red, green and blue (*r*,*g*,*b*) values, as acquired with the ColorRanger, over the entire ham slice and for both polarizer orientations. Thus, for each ham slice the (*r*_∥_,*g*_∥_,*b*_∥_) and (*r*_⊥_,*g*_⊥_,*b*_⊥_) values were obtained. The sum of (*r*_⊥_,*g*_⊥_,*b*_⊥_) indicated the lightness of the sample and an increase either in *r*_⊥_,*g*_⊥_ or *b*_⊥_, indicated increased lightness. An average of nine slices per formulation of cooked hams, were used in the image acquisition experiment.

### Supplementary Image Acquisition

**Fig 2:**
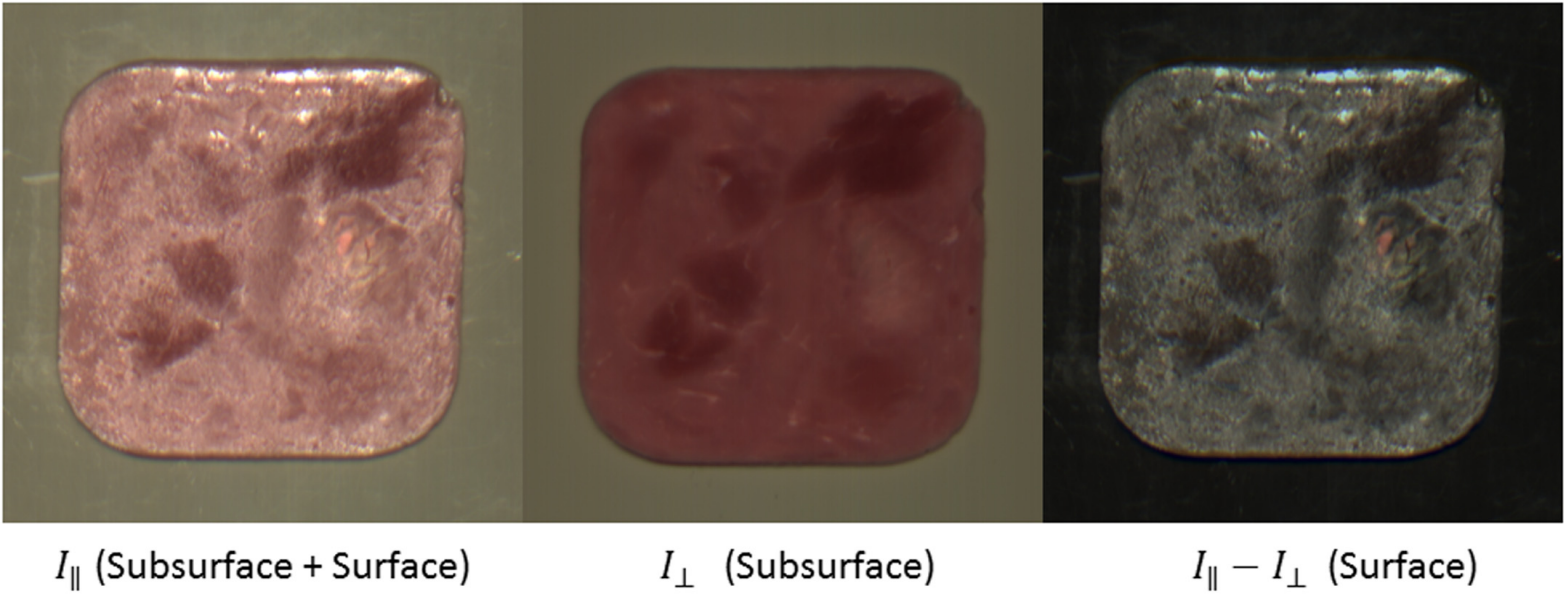
Images of a slice of ham, from supplementary Image Acquisition using parallel polarizers (left), crossed polarizers (middle) and the difference between the two (right).

Image acquisition of the hams used for the sensory evaluation was done with a polycarbonate window in the imaging setup, which unfortunately affected the polarization of the imaged light. This resulted in suboptimal images. Therefore, only the blue channel images (*b*_∥_ and *b*_⊥_) were used for analysis and a supplementary image acquisition was done to obtain more optimal images. Here, the polycarbonate window was replaced with an optical grade AR-coated BK7 glass window. A supplementary production of cooked hams was conducted in the same manner as for the main experiment, and three formulation of cooked ham (Na100, NaK80 and NaK60) were manufactured for the supplementary image acquisition, solely for validation of *the multimodal imaging acquisition system*. The formulation, production process, and storage were similar to the cooked ham preparation as described above. An example set of images is seen in Fig 2. The images in Fig 2 are included in order to provide the reader with a visual understanding of the possibilities of using multi-modal imaging of cooked ham with dual polarization states.

### Chemical analysis

**Water activity** (a_W_) was determined in cooked hams with a fast water activity-meter (GBX FAst/lab, Romans sur Isère Cedex, France). The **pH** measurements on light and dark muscle, in brine and cooked ham were carried out using a digital pH-meter WTW pH3110 (Weilheim, Germany) with a puncture electrode (WTW A 120513078, Weilheim, Germany). The **moisture** content was determined by drying three parallel samples of 5 g of minced cooked ham at 105°C for 24 hours [24]. **Water holding capacity (WHC)** was determined on minced cooked ham by low-speed centrifugation as described by Eide, Børresen [25] with a centrifugation force of 210 g. The WHC is expressed as the percentage of water retained in the mince after centrifugation for 5 min. The analyses were run in quadruplicate. **Sodium** contents in cooked hams were measured in an extract of the ham samples, using a Dual Star^TM^ pH/ISE Meter (Thermo Fisher Scientific, Waltham, MA, USA) with a Na-selective electrode (Ross® Sodium Ion Selective Electrode, Thermo Fisher Scientific, Waltham, MA USA). For preparing the extract, 7.5g of ham was homogenized in ultra-pure water using an Ultra-turrax T-25 D (IKA, Labortechnik, Staufen, Germany) at 9000 rpm for 1 min. Then, samples were warmed up to 90°C for 30 min, cooled down to room temperature, transferred to a volumetric flask and diluted to 200 mL with ultra-pure water. Finally, samples were filtered through a cellulose filter paper (Whatman n° 1, Whatman International Ltd., Maidstone, UK). The Na-selective electrode method was a modification of the Kivikari [26] method. In this study the direct calibration method was used, whereas Kivikari, used the known addition method. A calibration curve was made by using three standards of analytical-grade NaCl from (Merck KGaA, Darmstadt, Germany) and Sodium ionic strength adjustor (Thermo Fisher Scientific, Waltham, MA, USA) was added to all solutions to ensure that samples and standards had similar ionic strength.

The modified method of Grau and Hamm [27] was used to measure **expressible moisture** (**EM**) for cooked hams. Samples were punched out with a hollow drill (25 mm in diameter) from cooked slices of hams (15 mm thick). Each sample was placed in the middle of ten filter papers (Whatman No. 1) and pressed down with a flat-ended cylindrical plunger (80 mm diameter), by single compression test mode and a test speed of 0.8 mm/s until 50% compression of total height, using a Texture Analyzer T.A.XT2 (Stable Micro System, Surrey, U.K.). Expressible moisture was determined as the amount of water released per gram of meat and was expressed in percentage.

### Sensory evaluation

Descriptive Analysis (DA) was conducted by a trained sensory panel according to Generic Descriptive Analysis as described by [12]. All assessors (n = 9) were selected and trained in accordance with ISO 8586–1 (ISO, 1993) and the test was done in a sensory laboratory designed in accordance with ISO 8589 (ISO, 2007). Each assessor evaluated all samples using EyeQuestion for direct recording of data (v3.8.7, Logic8, Elst(Gld), The Netherlands). A list of 21 attributes was generated from a brainstorming session with the assessors. This list included attributes representing appearance (color-hue, color intensity, whiteness, color evenness, shiny, marbling, cohesiveness, visible moist on surface), odor (sour, pork meat, metal), taste/flavor (sourness, sweetness, saltiness, bitterness, pork meat, metal, after flavor) and texture (hardness, juiciness, tenderness). Attributes were evaluated using a continuous, non-structured scale ranging from no intensity (1) on the left to high intensity (9) on the right. The assessors had previous experience with analyses of meat products including ham and were calibrated on selected attributes in a pre-test. DA was performed during 6 sessions on one day, in which 8 different hams were served in two replicates (totally 16 samples). The hams were sliced by a machine to a weight of 16 ± 1 g, and each assessor got one slice served per sample. Appearance attributes were evaluated on the surface of the ham slice, while the texture was evaluated biting over a rolled slice. The serving order was randomized across all sessions. All samples were expectorated and unsalted crackers and lukewarm water was available for rinsing.

### Data analysis

The influence of the different levels of sodium on physicochemical properties was studied through one-way analysis of variance (ANOVA), (Minitab 16, Minitab Inc.) In cases where the effect was defined as significant (p<0.05), the means were compared using Tukeys test to find the significant difference between the samples containing different amounts of salt.

PanelCheck 1.3.2 (www.panelcheck.com) was used to evaluate the panel performance in the pre-test. For determination of sensory attributes discriminating between samples, a two-way ANOVA with product as a fixed factor, panelist as a random factor and product x panelist as an interaction factor was performed. Tukey’s Multiple Comparisons Test was applied to determine which products that was significantly different. The significance level was defined to p<0.05. (The ANOVA was run by SAS 9.2, SAS Institute, Inc., Cary, NS, USA).

## Results and Discussion

### Chemical analysis

The results of the chemical analyses of cooked ham at different salt/sodium levels are summarized in Table 2.

**Table 2:** Water holding capasity (WHC), moisture, pH, sodium, salt and expressible moisture of cooked ham prepared with different levels of salt at constant Na^+^: K^+^ mole ratio 3:1. The reference cooked ham contained nitrite salt (Na 100 Reference). In the rest of the formulas (NaK100, NaK80, NaK60, NaK40 and NaK20) the amount of salt was gradually decreased to 20% of the amount of salt in the reference ham. Mean values ± SD (n = 3).

Group	WHC (%)	Moisture (%)	pH	Sodium(g/100g)	Salt (g/100g)	Expressible moisture(%)
Na 100	78.45 ± 1.03^a^ ^b^ ^c^	74.58 ± 0.23^b^ ^c^	6.16 ± 0.02^a^ ^b^	1.35 ± 0.17^a^	3.38 ± 0.43^a^	0.96 ± 0.30^a^ ^b^ ^c^
NaK 100_1	82.57 ± 1.68^a^	73.63 ± 0.26^c^	6.15 ± 0.04^a^ ^b^	1.08 ± 0.01^b^	2.70 ± 0.03^b^	0.86 ± 0.16^a^ ^b^
NaK 100_2	80.59 ± 0.50^a^ ^b^	73.67 ± 0.15^c^	6.14 ± 0.04^a^	1.03 ± 0.04^b^ ^c^	2.58 ± 0.10^b^ ^c^	0.84 ± 0.16^a^
NaK 80	81.55 ± 1.13^a^ ^b^	74.40 ± 0.14^b^ ^c^	6.19 ± 0.02^a^ ^b^ ^c^ ^d^	0.92 ± 0.01^c^ ^d^	2.25 ± 0.03^c^ ^d^	1.07 ± 0.23^d^ ^e^
NaK 60_1	80.83 ± 0.16^a^ ^b^	73.83 ± 0.19^a^	6.21 ± 0.02^b^ ^c^ ^d^ ^e^	0.82 ± 0.01^d^ ^e^	2.05 ± 0.03^d^ ^e^	1.55 ± 0.39^d^ ^e^
NaK 60_2	79.54 ± 0.88^a^ ^b^ ^c^	75.14 ± 0.18^a^ ^b^	6.21 ± 0.02^b^ ^c^ ^d^	0.81 ± 0.01^d^ ^e^	2.03 ± 0.03^d^ ^e^	1.12 ± 0.17^a^ ^b^ ^c^ ^d^
NaK 40	76.31 ± 0.25^c^ ^d^ ^e^	75.43 ± 0.28^a^ ^b^	6.24 ± 0.02^c^ ^d^ ^e^ ^f^	0.69 ± 0.01^e^ ^f^	1.73 ± 0.03^e^ ^f^	1.49 ± 0.23^c^ ^d^ ^e^
NaK 20	72.71 ± 1.83^d^ ^e^	75.40 ± 0.46^a^ ^b^	6.25 ± 0.01^d^ ^e^ ^f^	0.55 ± 0.01^f^	1.38 ± 0.03^f^	1.96 ± 0.35^e^
p-value	0.000	0.000	0.000	0.000	0.000	0.000

^a-g^ Different upper-case letters within a column indicate significant differences (p<0.005) between different groups. Means that do not share a common letter are significantly different.

### Sodium content

The salt contents (%) in the cooked ham were calculated as measured Na^+^-content multiplied with 2.5. The measured sodium content in the reference ham (Na 100 added as Na^+^—ions only) was 1.35 g Na^+^/100 g, this result was slightly higher than estimated in the formulation of the sample, 1.24 g Na^+^/100g. The rest of the hams, were prepared with different levels of total salt including a constant Na^+^:K^+^ mol ratio of 3:1, and the measured sodium content were in good agreement with the estimated in the formulation.

### Water holding capacity (WHC)

As expected, a decrease in WHC was observed in hams where the salt content was reduced by 60% (NaK40) and 80% (NaK20) (on molar basis), corresponding to a measured salt content of 1.73 and 1.38% salt, respectively. These results are in accordance with Hamm (1972) and Offer & Knight (1988) who found that sodium chloride increases the water-binding of meat [28, 29]. Albarracín, Sánchez [30] explained that increased WHC in meat, resulting from increasing salt content, is due to the anions (Cl^-^) preferentially binding to protein molecules. The moisture content in hams added a mixture of sodium and potassium salt without reduction of the total salt, 2.70% salt (NaK100) was significant lower than in cooked ham containing 1.38% salt (NaK20).

### pH

The pH in the raw material was within the range of 5.5–5.8. These pH values are similar to typical ultimate pH values in Norwegian pork [31]. The pH of the hams increased from pH 6.15 ± 0.02 to 6.25 ± 0.01 with decreasing sodium content. The pH in hams with the lowest levels of sodium, 1.73% salt (NaK40) and 1.38% salt (NaK20), were significant higher than the reference containing 3.38% salt (Na100) and the cooked ham with the mixture of sodium and potassium salt (NaK100). These findings are in accordance with Puolanne, Ruusunen [32], who found an average decrease, about 0.1 pH-units/%-units of salt, in cooked sausages.

### Expressible moisture

The expressible moisture increased with decreasing salt/sodium content, and those hams containing 1.38% salt (NaK20) had significantly higher expressible moisture than those containing 3.38% salt (Na 100).

### Water activity (a_w_)

The a_w_ decreased slightly from 0.97 to 0.96 with increasing salt content, although the differences were not significant (data not shown). According to Mossel, Corry [33], such levels of a_w_ in cooked hams were too high to represent a hurdle for unwanted spoilage- and pathogenic microorganisms.

Compared to the reference ham (Na100), a replacement of Na^+^-ions by 25% of K^+^-ions gave no significant changes in WHC, moisture, pH or expressible moisture. This replacement results in a total sodium content of approximately 1.1 g /100g cooked ham. This finding is in accordance with Zanardi, Ghidini [34] who found no effects on pH and water activity in reduced sodium when replacing (Na^+^) by a mixture of KCl, CaCl_2_ and MgCl_2_ in Cacciatore salami compared to the traditional recipe.

### Descriptive sensory profile

Table 3 gives an overview of the mean values for the significant sensory attributes plus the attributes whiteness and shiny, as appearance attributes were most relevant for data from vision analysis. The table shows significance for 8 out of a total of 21 sensory attributes.

**Table 3:** Sensory properties in the cooked hams.

	Apperance				Taste and odour				Texture	
Group	Colour hue	Cohesiveness	Whiteness	Shiny	Salty taste	Metal flavour	Metal odour	After taste	Tenderness	Hardness
Na 100	6.67^a^ ^b^ ^c^	6.97^a^ ^b^	5.61^a^ ^b^	3.71	6.18^a^ ^b^ ^c^ ^d^	4.74^a^ ^b^ ^c^	4.94^a^ ^b^ ^c^	5.92^a^ ^b^ ^c^	5.36^a^	4.78^a^ ^b^
NaK 100_1	6.84^a^ ^b^ ^c^	7.12^a^ ^b^	5.59^a^ ^b^	4.21	6.53^a^ ^b^	5.12^a^	5.19^a^	6.21^a^ ^b^	5.02^a^	5.03^a^
NaK 100_2	6.97^a^ ^b^	7.42^a^	5.49^a^ ^b^	3.76	6.58^a^	4.83^a^ ^b^ ^c^	4.91^a^ ^b^ ^c^	6.36^a^	5.11^a^	4.91^a^ ^b^
NaK 80	6.97^a^ ^b^	7.18^a^	5.61^a^ ^b^	4.09	6.18^a^ ^b^ ^c^ ^d^	4.92^a^ ^b^	4.84^a^ ^b^ ^c^	6.14^a^ ^b^ ^c^	5.09^a^	5.02^a^
NaK 60_1	6.77^a^ ^b^ ^c^	6.94^a^ ^b^	5.79^a^ ^b^	3.32	5.55^b^ ^c^ ^d^ ^e^	4.76^a^ ^b^ ^c^	4.94^a^ ^b^ ^c^	5.52^b^ ^c^	5.40^a^	4.73^a^ ^b^
NaK 60_2	6.49^a^ ^b^ ^c^ ^d^	7.27^a^	5.72^a^ ^b^	3.98	5.26^d^ ^e^	4.64^a^ ^b^ ^c^	5.08^a^ ^b^	5.54^b^ ^c^	5.88^a^ ^b^ ^c^	4.50^a^ ^b^ ^c^
NaK 40	6.77^a^ ^b^ ^c^	6.66^a^ ^b^ ^c^	5.91^a^ ^b^	3.76	4.90^e^ ^f^	4.34^a^ ^b^ ^c^	4.63^a^ ^b^ ^c^	5.73^a^ ^b^ ^c^	6.13^a^ ^b^ ^c^	4.06^c^ ^d^
NaK 20	5.99^d^	6.01^b^ ^c^	6.20^a^	3.36	4.06^f^	4.03^b^ ^c^	4.28^b^ ^c^	4.73^d^	6.83^b^ ^c^	3.58^d^ ^e^
p-value	0.0010	0.0013	n.s.	n.s.	<0.0001	0.0065	0.0098	<0.0001	0.0001	<0.0001

^a-f^ Different upper-case letters within a column indicate significant differences (p<0.005) between different groups. Means that do not share a common letter are significantly different. Non-significant: n.s.

### Appearance

Compared to the reference ham (Na100), a replacement of Na^+^ ions by K^+^ ions of 25% and reduction to 60% (NaK40) of total salt had no effect on the color hue. However, a reduction of total salt content by 80% and replacement of Na^+^ ions by K^+^ ions of 25% (NaK20) showed a significant lower color hue. There were significant differences between samples for cohesiveness, but there were no clear correlation between the salt content and the cohesiveness score. A possible explanation might be the test production. In small scale test production without vacuum, it can be difficult to avoid air and small gaps between the muscle pieces.

### Taste, flavor and odor

A reduction to 40% (NaK60) of total salt, corresponding to 2.04% salt, was possible without significantly influencing the salty taste. The changes in saltiness in this experiment correspond to another low-salt ham study, Ruusunen, Särkkä-Tirkkonen [5] found that hams with 1.4% salt were less salty than those containing 1.7–2.6% salt. The reduced saltiness can be explained by the reduced content of Cl^-^—anions in the cooked ham since Cl^-^ anions have an effect on the receptor cells and consequently on the perception of salt taste [35]. There were no significant differences in metal flavor and metal odor except for a higher value for metal flavor for one of the parallels of cooked ham containing the highest amount of potassium chloride (NaK100_1) compared to the cooked ham containing the lowest salt content (NaK20). These results indicate that a three to one replacement of Na^+^ ions with K^+^ ions might be an acceptable level, regarding the issue of bitter (no significant differences between samples) and metallic flavor (NaK20 significant different from the other samples). Previous studies have shown that KCl may induce a bitter taste at high concentrations. In dry-cured loins [9] and fermented sausages [10], the maximum level was 40% replacement of sodium with potassium. In a study of cooked ham they showed that 2.0% NaCl had a higher score than a 50% replacement with KCl [11]. Another study found that it was possible to replace one-third of NaCl with KCl without altering the sensory properties of smoked salmon [36]. Those results show that the maximum level of KCl replacement may be dependent on product type.

Table 3 shows that a reduction to 60% (NaK40) of total salt had no effect on the after taste. However, a reduction of total salt content by 80% and replacement of Na^+^ ions by K^+^ ions by 25% (NaK20) gave a less pronounced aftertaste compared to all other hams. This can be explained by the fact that salt has a flavor enhancing effect in meat products [37], and the salt content can also effect on biochemical and enzymatic processes that in turn may impact flavor and/or structure of a product [30]. These findings are in accordance with Ruusunen, Vainionpää [38], who found that ground meat patties with salt content down to 0.6% NaCl, had weak flavor intensity.

### Texture

Compared to the reference ham (Na100), a reduction down to 60% (NaK40) of total salt, corresponding to 1.73% salt, had no effect on tenderness or hardness in the cooked hams. However, a reduction of total salt content by 80% and replacement of Na^+^ ions by K^+^ ions of ¼ (NaK20), corresponding to a salt content of 1.38%, gave a more tender and less hardness on cooked ham. These results were as expected and in accordance with Ruusunen, Vainionpää [39], who found that more added salt increased the firmness in Bologna type sausages at 1.10, 1.35 to 1.60% added salt, Sofos [40] found that a reduction in salt content of more than 20% (<2.0% NaCl) in Frankfurters resulted in softer and less firm texture. It is important to keep in mind that the effects of proteins may differ in cooked ham where small pieces of lean meat are connected together whereas sausages have an emulsified fat protein network. In both cases the solubility and swelling of the myosin are nevertheless important. In cooked ham, molecular bonds are able to form inside the exudate matrix (gel cohesion) and between the exudate and the muscle [20], and when the salt concentration is reduced, the protein extractability is limited due to the solubility of the myosin [28, 29].

Compared to the reference ham (Na100), a replacement of Na^+^-ions by K^+^-ions of 25% gave no significant changes in sensory profile attributes. This replacement corresponds to a total sodium content of approximately 1.1 g /100 g cooked ham. These findings are in accordance with other studies [9–11, 36].

### Multimodal machine vision system

The main imaging experiment, using the multimodal machine vision system, was done using the same hams as with the chemical and sensorial measurements.

**Fig 3:**
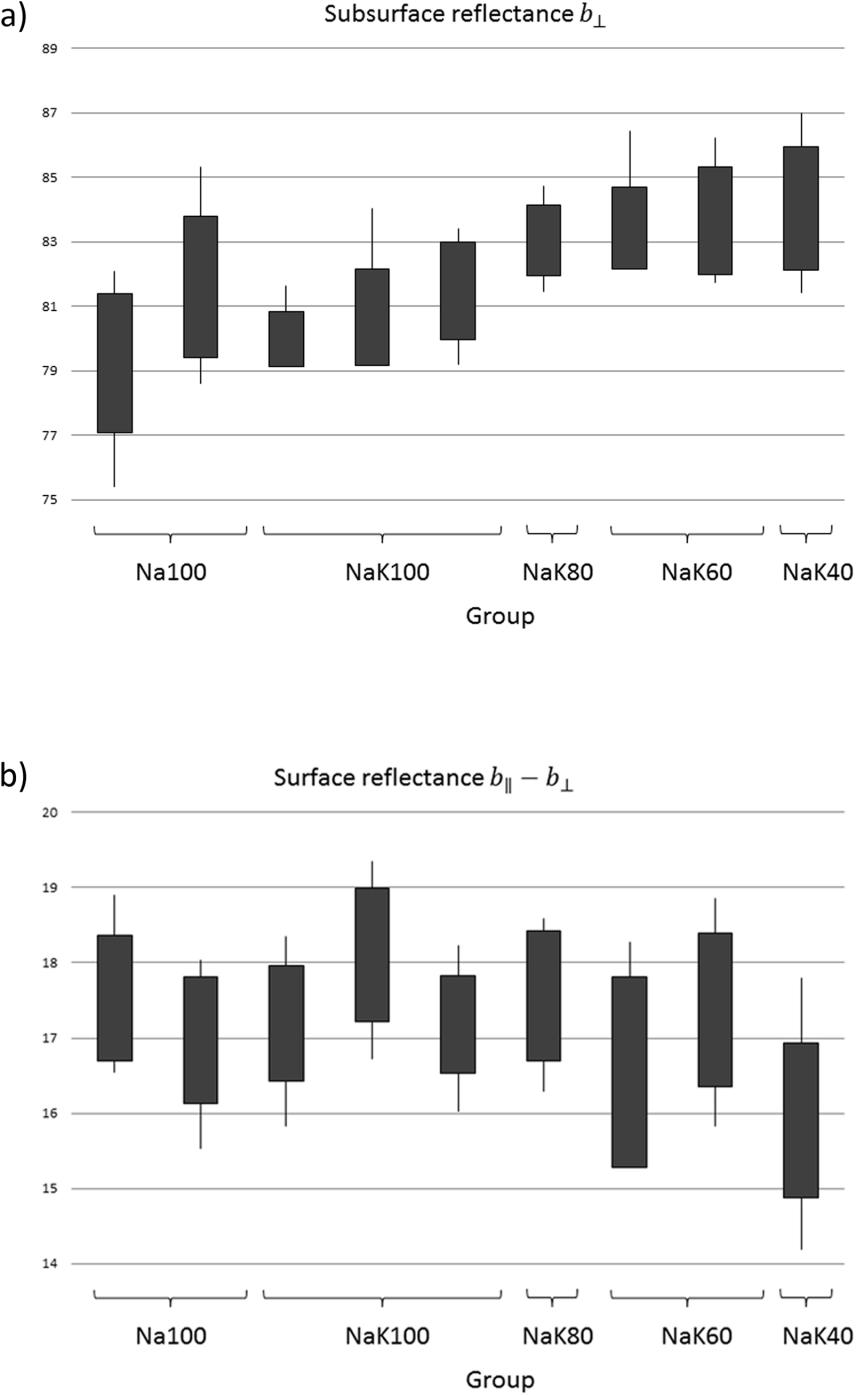
A, Mean subsurface reflectance *b*_⊥ for the hams with varying salt content. B, Mean surface reflectance *b*_∥−*b*_⊥ for the hams with varying salt content.

Machine vision was used to objectively study changes in color and texture in the surface. The change in lightness, as a function of reducing the salt content, is clearly seen in Fig 3A.

The intensity of the subsurface backscattered reflected blue light *b*_⊥_ shows a clear increase as the salt content is reduced. This is confirmed by sensorial evaluation, indicating an increase in whiteness and decrease in color-hue with decreased salt content. A possible explanation for this behavior might be the decreased swelling of the salt soluble proteins and a weaker heat induced gel in cooked ham with 1.38% salt (NaK20) as discussed in above in section 3.3. Due to this theory, the surface of the cooked ham with less salt might be rougher, and the interaction of light will change, as has been shown previously [13]. Another explanation for the increased whiteness due to decreased salt content may be that salt affects the formation of heat stable nitric oxide myoglobin, the "cured meat color" [41].

A reduction in surface shininess, as measured by *b*_∥_−*b*_⊥_, using imaging, was only weakly linked to reduction in salt content, Fig 3B. Despite the challenges in the imaging setup in the main experiment, the improvements from the supplementary experiment in Fig 2 show that imaging with two polarization orientations can provide images that potentially can quantify subtle changes in visual appearance.

Since only simple features were used, namely the (*r*_∥_,*g*_∥_,*b*_∥_) and (*r*_⊥_,*g*_⊥_,*b*_⊥_) values computed as the mean over each slice, there is a greater potential in dual-polarization imaging if more advanced features such as those described in the literature [13–15, 17, 19] would be applied to dual-polarization images using the multimodal machine vision system described in this paper. Automatic segmentation, between the dark and light portions of the meat, may also help improve the quantification of the visual appearance characteristics, similar to how that human sensorial evaluators observe using their combined vision and decision processes.

## Conclusions

Compared to the reference ham (3.4% salt), a replacement of 25% Na^+^-ions by K^+^-ions gave no significant changes in WHC, moisture, pH, expressed moisture, the sensory profile attributes or the surface lightness and shininess. However, a reduction of salt content by 60% and 80% on molar basis or more (down to 1.7–1.4% salt) led to a decrease in WHC and an increase in expressible moisture. The salt reduction had highest influence on the sensory attributes salty taste, after taste, tenderness, hardness and color hue. To summarize, a reduction to 40% of total salt, corresponding to 2.04% salt, was possible without significantly influencing the salty taste. Further reduction down to 1.4% salt led to increased softness and reduced hardness in the cooked hams. When reduction of salt by more than 60% (down to 1.7% salt) together with a replacement of 25% Na^+^-ions by K^+^-ions, the low-salt ham manufacture procedure has to be modified. The multimodal machine vision system showed changes in lightness, as a function of reduced salt content.

## References

[1] He FJ, MacGregor GA. Effect of modest salt reduction on blood pressure: a meta-analysis of randomized trials. Implications for public health. J Hum Hypertens. 2002;16(11):761–70. 12444537 10.1038/sj.jhh.1001459

[2] WHO. Reducing salt intake in populations. Report of a WHO Forum and Technical Meeting. World Health Organization, Paris, 2006.

[3] The Norwegian Directorate of Health. Strategy for the reduction of salt intake in the population Recommendation from the National Nutrition Council. In: The Norwegian Directorate of Health, editor. Available: http://helsedirektoratet.no/folkehelse/ernering/strategier-og-satsninger/Documents/saltstrategi.pdf: The Norwegian Directorate of Health; 2011.

[4] FSAI. Food Safety Authority of Ireland, Salt and health: review of the scientific evidence and recommendationsfor public policy in Ireland. 2005.

[5] Ruusunen M, Särkkä-Tirkkonen M, Puolanne E. Saltiness of coarsely ground cooked ham with reduced salt content. Agricultural and food science in Finland. 2001;10:27–31.

[6] Pietrasik Z, Gaudette NJ. The impact of salt replacers and flavor enhancer on the processing characteristics and consumer acceptance of restructured cooked hams. Meat Sci. 2014;96(3):1165–70. 10.1016/j.meatsci.2013.11.005 24334036

[7] Breslin PAS, Beauchamp GK. Suppression of Bitterness by Sodium: Variation Among Bitter Taste Stimuli. Chem Senses. 1995;20(6):609–23. 8788095 10.1093/chemse/20.6.609

[8] Olson D. Salt for processing probably can be cut by only one quarter. The National Provisioner. 1982;7–10.

[9] Gou P, Guerrero L, Gelabert J, Arnau J. Potassium chloride, potassium lactate and glycine as sodium chloride substitutes in fermented sausages and in dry-cured pork loin. 1996(0309–1740 (Print)). 10.1016/0309-1740(95)00017-8 22060300

[10] Gelabert J, Gou P, Guerrero L, Arnau J. Effect of sodium chloride replacement on some characteristics of fermented sausages. Meat Sci. 2003;65(2):833–9. 10.1016/S0309-1740(02)00288-7 22063446

[11] Frye CB, Hand LW, Calkins CR, Mandigo RW. Reduction or Replacement of Sodium Chloride in a Tumbled Ham Product. J Food Sci. 1986;51(3):836–7.

[12] Lawless HT, Heymann H. Sensory evaluation of food—principles and practices (2nd ed.). New York: Springer; 2010.

[13] Iqbal A, Valous NA, Mendoza F, Sun D-W, Allen P. Classification of pre-sliced pork and Turkey ham qualities based on image colour and textural features and their relationships with consumer responses. Meat Sci. 2010;84(3):455–65. 10.1016/j.meatsci.2009.09.016 20374810

[14] Jackman P, Sun DW, Du CJ, Allen P. Prediction of beef eating qualities from colour, marbling and wavelet surface texture features using homogenous carcass treatment. Pattern Recognition. 2009;42(5):751–63.

[15] Jackman P, Sun DW, Allen P. Prediction of beef palatability from colour, marbling and surface texture features of longissimus dorsi. J Food Eng. 2010;96(1):151–65. 10.1016/j.meatsci.2009.10.013 20374825

[16] Valous NA, Mendoza F, Sun D-W, Allen P. Texture appearance characterization of pre-sliced pork ham images using fractal metrics: Fourier analysis dimension and lacunarity. Food Res Int. 2009;42(3):353–62.

[17] Chandraratne MR, Samarasinghe S, Kulasiri D, Bickerstaffe R. Prediction of lamb tenderness using image surface texture features. J Food Eng. 2006;77(3):492–9.

[18] Alçiçek Z, Balaban MÖ. Development and application of “The Two Image” method for accurate object recognition and color analysis. J Food Eng. 2012;111(1):46–51.

[19] Mendoza F, Valous NA, Allen P, Kenny TA, Ward P, Sun D-W. Analysis and classification of commercial ham slice images using directional fractal dimension features. Meat Sci. 2009;81(2):313–20. 10.1016/j.meatsci.2008.08.009 22064169

[20] Bombrun L, Gatellier P, Carlier M, Kondjoyan A. The effects of low salt concentrations on the mechanism of adhesion between two pieces of pork semimembranosus muscle following tumbling and cooking. Meat Sci. 2014;96(1):5–13. 10.1016/j.meatsci.2013.06.029 23896131

[21] Aaslyng MD, Vestergaard C, Koch AG. The effect of salt reduction on sensory quality and microbial growth in hotdog sausages, bacon, ham and salami. Meat Sci. 2014;96(1):47–55. 10.1016/j.meatsci.2013.06.004 23896136

[22] Mathiassen JR, Misimi E, Bondø M, Veliyulin E, Østvik SO. Trends in application of imaging technologies to inspection of fish and fish products. Trends in Food Science & Technology. 2011;22(6):257–75.

[23] Regulation EU no 1169/2011 of the European Parliament and the council of 25^th^ October 2011. In Official Journal of the European Union L 304/18 22.11.2011.

[24] AOAC. Official Methods of Analysis of the Association of Official Analytical Chemists. 15th ed. Arlington, Virgina: AOAC; 1990.

[25] Eide O, Børresen T, Strøm T. Minced Fish Production From Capelin (Mallotus villosus). A New Method for Gutting, Skinning and Removal of Fat from Small Fatty Fish Species. J Food Sci. 1982;47(2):347–9.

[26] Kivikari R. Analysis of sodium in meat products using an Na-selective eletrode. (In Finnish). Proc. of Meat Day Seminar 1996, Nr, 536. Proc. of Meat Day Seminar 1996. 1996.

[27] Grau R, Hamm R. Eine einfache Methode zur Bestimmung der Wasserbindung im Muskel. Die Naturwissenschaften. 1953;40(1):29–30.

[28] Hamm R. Koloidchemie des Fleisches: Parey, Berlin; 1972.

[29] Offer G, Knight P. The structural basis of water-holding in meat DimS-R.A. Lawrie, editor. Capters 3–4: London: Elsevier Applied Science; 1988.

[30] Albarracín W, Sánchez IC, Grau R, Barat JM. Salt in food processing; usage and reduction: a review. Int J Food Sci Technol. 2011;46(7):1329–36.

[31] Gjerlaug-Enger E, Aas L, Ødegård J, Vangen O. Genetic parameters of meat quality traits in two pig breeds measured by rapid methods. Animal. 2010;4(11):1832–43. 10.1017/S175173111000114X 22445144

[32] Puolanne E, Ruusunen MH, Vainionpää JI. Combined effects of NaCl and raw meat pH on water-holding in cooked sausage with and without added phosphate. Meat Sci. 2001;58(1):1–7. 22061912 10.1016/s0309-1740(00)00123-6

[33] Mossel DAA, Corry JET, Struijk CB, Bair RM. Essentials of the Microbiology of Foods A Textbook for Advanced Studies: John Wiley & Sons, Chichester, England; 1995.

[34] Zanardi E, Ghidini S, Conter M, Ianieri A. Mineral composition of Italian salami and effect of NaCl partial replacement on compositional, physico-chemical and sensory parameters. Meat Sci. 2010;86(3):742–7. 10.1016/j.meatsci.2010.06.015 20663614

[35] Murphy C, Cardello AV, Brand JG. Tastes of fifteen halide salts following water and NaCl: Anion and cation effects. Physiology & Behavior. 1981;26(6):1083–95. 7280070 10.1016/0031-9384(81)90213-4

[36] Almli V, Hersleth M. Salt replacement and injection salting in smoked salmon evaluated from descriptive and hedonic sensory perspectives. Aquaculture International. 2013;21(5):1091–108.

[37] Desmond E. Reducing salt: A challenge for the meat industry. Meat Sci. 2006;74(1):188–96. 10.1016/j.meatsci.2006.04.014 22062728

[38] Ruusunen M, Vainionpää J, Lyly M, Lähteenmäki L, Niemistö M, Ahvenainen R, et al Reducing the sodium content in meat products: The effect of the formulation in low-sodium ground meat patties. Meat Sci. 2005;69(1):53–60. 10.1016/j.meatsci.2004.06.005 22062639

[39] Ruusunen M, Vainionpää J, Puolanne E, Lyly M, Lähteenmäki L, Niemistö M, et al Effect of sodium citrate, carboxymethyl cellulose and carrageenan levels on quality characteristics of low-salt and low-fat bologna type sausages. Meat Sci. 2003;64(4):371–81. 10.1016/S0309-1740(02)00178-X 22063117

[40] Sofos JN. Effects of Reduced Salt (NaCl) Levels on Sensory and Instrumental Evaluation of Frankfurters. J Food Sci. 1983;48:1692–9.

[41] Honikel K-O. The use and control of nitrate and nitrite for the processing of meat products. Meat Sci. 2008;78(1–2):68–76. 10.1016/j.meatsci.2007.05.030 22062097

